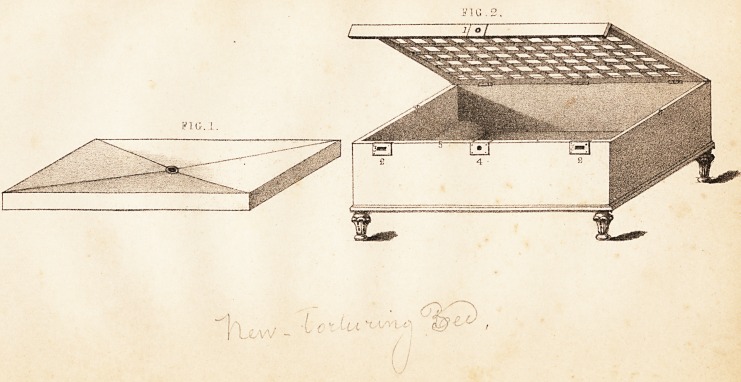# Dr. Williams on Insanity
†"Insanity; its Causes, Prevention, and Cure, &c." By Jos. Williams, M.D. London: Churchill, 1852.


**Published:** 1852-07-01

**Authors:** 


### Art. II.—DR. WILLIAMS ON INSANITY.†

Dr. Williams has the honour of having written the most *recent* monograph on Insanity. As his work has been extensively advertised as the "Lord Chancellor's Prize Essay," we opened the volume with the assurance that we should find in its pages a record of both novel and valuable views relative to the pathology of the brain, and the therapeutics of insanity. We regret to say that we have been grievously disappointed. It would have afforded us much satisfaction to have had an opportunity of recommending this work to the favourable notice of our readers; but a stern sense of critical justice compels us to withhold from Dr. Williams's volume the stamp of our approbation. The first

† "Insanity; its Causes, Prevention, and Cure, &c." By Jos. Williams, M.D. London: Churchill, 1852.

edition of this work was published under the title of "An Essay on the Use of Narcotics and other Remedial Agents calculated to produce Sleep, in the Treatment of Insanity;" and for this essay a premium, which the present highly distinguished Lord Chancellor, when presiding over the Irish Court of Chancery, placed at the disposal of the President and Fellows of King and Queen's College of Physicians, was awarded. Of this we have no right to complain. Dr. Williams has re-written the "prize essay," and has published it under a totally different title, still announcing it to the public as the "Lord Chancellor's Prize Essay on Insanity." We do not wish to make any observations personally annoying or offensive to the author; but we ask, whether he is justified in so advertising his present work? Sir Edward Sugden's prize was far the best essay on a specific form of treating insanity by means of *narcotics;* Dr. Williams's present volume is entitled "Insanity; its Causes, Prevention, and Cure, including Apoplexy, Epilepsy, and Congestion of the Brain!" This can no more be considered *the* prize essay of Lord St. Leonards, than the last volume of the "Lancet," or the "Psychological Journal." It is quite a misnomer, and we regret that Dr. Williams has been so ill-advised as to designate it as such. If this practice be allowed, much mischief to the cause of legitimate literature will inevitably ensue. A premium is awarded for a given essay, selected perhaps from a number of MSS. sent in for competition; a particular essay is approved of, and its author carries off the prize. The matter of the essay is unobjectionable; it is supposed to contain no false facts, wrong deductions, or mischievous doctrines, and it is sent forth to the world stamped by the authority of those selected to adjudicate in the matter. A few years elapse, and the little prize essay swells out into a huge volume, containing perhaps not fifty pages of the original essay, but several hundred pages of *new matter*. Is it fair that this new—entirely new—work should be announced to the public as the original prize essay? The new work may contain very questionable matter—false data and mischievous conclusions, which the adjudicators might greatly disapprove of: we say this is possible, and therefore it is unfair to them that it should be published as the original prize essay. On *principle* we object to this proceeding, and we feel it our duty to direct attention to the fact. Dr. Williams may advertise his *original essay*, which, in all its features, is very different from the volume under consideration, as "Sir Edward Sugden's Prize Essay;" but he cannot *honestly* put his *present* volume forward as the one submitted to the President and Fellows of King and Queen's College of Physicians, and to which the Irish Lord Chancellor's prize was awarded. Having made these preliminary remarks, we proceed to consider the contents of the volume itself. Having perused Dr. Williams's work attentively, with every wish to speak favourably of it, we are compelled to withhold from the essay our approbation. It is composed of vapid nonentities and common-place truisms, interlarded with hackneyed quotations from Dr. *this* and Dr. *that*, and Mr. A. and Mr. B., without an observation of the author's which may be termed either novel or original. We will not pretend to divine the motives which may have led Dr. Williams thus to rush into print.

> "I too can write; and once upon a time
> I poured along the town a flood of rhyme,
> A schoolboy trick, unworthy praise or blame;
> I printed—older children do the same."

Dr. Williams prefaces his essay with a few metaphysical reflections and observations. We shall content ourselves with making only a few quotations from this portion of the work. After announcing the important fact that "the mind is an immortal and immaterial entity," Dr. Williams ventures, with a degree of moral courage which we are bound to commend, to enumerate a great metaphysical truth which he has had the good fortune to discover. It has remained for that gentleman to settle beyond, we trust, all future cavil or dispute, the long vexed question with reference to the distinction between *mind* and *soul.* All honour to Dr. Williams, and to the county which gave him birth! The intellect, says this great metaphysician, "is the mind, whilst it occupies its earthly tenement; but having left it, it is called *soul.*" By *whom* it is so called the author omits to state. The Doctor does not venture to explain by what process of induction he has arrived at a knowledge of this great fact; he announces the discovery with all the modesty which usually accompanies genius, simply observing in a foot note, that "I shall remain satisfied with this dogmatical statement, as it is my intention at an early period to discuss and illustrate this polemical and psychological subject in one of the Reviews." We await with feelings of intense interest this promised illustration, and until we have the pleasure of perusing Dr. Williams's exposition, we must, we presume, take for granted the metaphysical question as settled. Metaphysicians, and theologians, must for the future cease to talk of the soul until after the extinction of vitality; the soul having no existence during life, it being nothing but an off-shoot or prolongation of mind. The discovery of Dr. Williams must tend to revolutionize the whole of metaphysical science! The introductory chapter of the work before us is replete with valuable apothegms. For example: "*The soul or mind can never perish.*" "*Or mind.*" How does the Doctor reconcile this expression with the previously enunciated doctrine relative to the soul? "*Man is a rational creature,*"—"*by reason we distinguish right from wrong;*" the "*highest faculty is*

*termed self-inspection or reflection.*" This is the first time we have heard reflection, or, as Dr. Williams classically designates it, "self-inspection," termed the "highest faculty of the mind." Whatever is perceived, is called an idea *in its enlarged sense.*" What is it, we ask, in its contracted or limited sense? "*Imagination is purely intellectual.*" Who ever dreamt of its being otherwise? "*Imagination enables us to conceive, compose, and form new ideas;*" such is not the province *usually* assigned to this faculty. Certainly *conception* cannot properly be deemed as one of its attributes. "*Imagination is often one of the most dangerous gifts* (?) *a man can possess, impairing his judgment and weakening his attention.*" This may be said of any faculty of the mind if not properly regulated, disciplined, and controlled. If not *abused*, the imagination is a most essential, useful, and important faculty of the mind; we do not conceive how it can be justly designated "as one of the *most dangerous gifts* a man can possess," or how it can be supposed to "impair the judgment," or "weaken the attention." After detailing various definitions of insanity, Dr. Williams ventures to give his own test of unsoundness. "That man," he says, "may be said to be insane who has no control over his thoughts and actions." We sincerely hope for the peace of mind and reputation of the Doctor, that he will never propound *such a definition of insanity*, should he ever have the fortune or *mis*-fortune to take his place in the witness box. If the loss of control over the thoughts and actions constitute insanity, we tremble for a large class of Her Majesty's subjects, at present permitted to roam in undisturbed possession of their liberty, and left without hindrance to manage themselves and their property. Let Dr. Williams be assured, that a definition of insanity is a hazardous experiment. "The most simple deviation from insanity is *usually termed* eccentricity." (p. 8.) By whom is it so termed? We do not recognise this "*simple deviation.*" Eccentricity may, and often does exist to a considerable extent, the mind being *sane*. Does Dr. Williams deny the fact? "A man," says our author, may be eccentric in dress, in manners, in habits, or he may draw inferences at variance with the opinions of a sound judgment, being different from those of any sensible person; now, *such affectation* as *this is apt to grow*," and Dr. Williams, therefore, suggests that the "affectation" leading to eccentricity "should be checked in its earliest development." After alluding to the fact that "lunatics are often very vain;" that they are "fond of assuming high characters;" Dr. Williams with great *naïveté* observes, "it is right here to mention that Gall and his disciples believed that these assumptions of different characters depend upon various portions of the brain being affected." We beg to call particular attention to the distinction which Dr. Williams draws between a "*weak*" and an "*insane*"

mind. "A person of merely weak mind," says our author, "although he may be very eccentric and foolish, yet when his errors are pointed out by another, *he sees and admits them*." How fortunate it would be for society if such were the fact. The folly of the merely "weak mind," is often exhibited in an obstinate and tenacious adherence to absurd conceits. "Though thou shouldst bray a fool in a mortar among wheat with a pestle, yet will not his foolishness depart from him." Again, our author observes,—"*Unsoundness of mind* consists in a *morbid condition of intellect*, or loss of reason, coupled with an incompetency of the person to manage his own affairs," (p. 14.) This is tantamount to a declaration that *unsoundness of mind* is *insanity*, and *insanity* is a *morbid condition of the intellect;* this explanation, lucid as it may appear to the Doctor, leaves us as much as ever in the dark.

It is important to notice that, among the symptoms exhibited by those "predisposed to insanity," to which the author refers, "is *a remarkable fondness of showing off* (?), *and reciting, and spouting*." We would have some of our amateur theatrical friends bear this in mind. In the celebrated commission of lunacy in the case of Mr. Davis, one of the arguments in proof of his insanity was, that he "spouted" and "recited" Shakespeare with insane elevation of voice, and with a morbid vehemence and warmth of gesticulation. It was when giving evidence in this inquiry, that Dr. Haslam, much to the amusement of the court, and Mr. Davis's celebrated counsel, Mr. H. Brougham (now Lord Brougham), talked of the alleged lunatic labouring under a "*delusion of manner*." We were present in court at the time, and well recollect the ludicrous effect which this remark had upon all present. For the future, we presume, we shall be justified in talking of the "delusion of showing off," and of "reciting and spouting." In the chapter on *Suicide*, we find nothing worthy of notice. We are informed that "in France, where foolish lovers together commit this deed, (how pathetic!) they often meet a united death in the fumes of carbonic acid gas." In the vulgar tongue, they destroy themselves by ignited charcoal. Again, the author communicates to us the novel and important information, that, "persons who commit suicide *have often insane relations*, and there can be *no doubt* that suicide is in some instances *hereditary*." We trust, after this announcement, the fact of the hereditary character of the suicidal disposition will be no longer questioned. The whole chapter on "Suicide," and "Melancholia," is replete with common-place observations, to be found in every elementary work on insanity. There does not occur one remark in these chapters quotable on account of its originality. For example, we are gravely informed, as if the author had lighted upon a great psychological fact, that "many persons are unable to look down from any great height without feeling an inclination to throw themselves down." Then follows the author's *rationale* of this very singular and startling fact. "This (he says) does not arise from *giddiness*, but seems to depend upon some *peculiar fascination*." After a pointless and senseless tirade against theatrical performances, the author discusses, in *five* pages, the important and comprehensive subject of "religious insanity," in which we find the following astute observation:—"Persons afflicted with religious insanity *sometimes require* watching (an important admission,) as they occasionally become dangerous, hearing *whisperings* (?), which tell them to take the lives of their infants," &c. When speaking of cases of "*moral insanity*," in which, by the by, Dr. Williams erroneously says "there is *no* illusion, *no* hallucination," our author observes, "those individuals (the morally insane) feel inclined to break china (so do some sane women, when irritated by their husbands), dash down girandoles, or crack any small objects of vertu." Heaven preserve a number of her Majesty's male and female subjects, disposed to "break china," "dash down girandoles," &c., "crack small objects of vertu," or their own heads, if they come within the "long range" of those who thus define moral insanity! The author's account of what is termed "moral insanity," is excessively meagre; many of the more peculiar and pathognomonic features of the disorder are entirely omitted or cursorily passed over. Take, for instance, Dr. Williams' description of the disease. "Some persons utter words they do not wish, being unable to control or direct them, (so do those who are said to be 'intellectually insane,') yet knowing them not to be correct. The same has occurred in writing: thus in drawing a cheque he has begun correctly enough; but in continuing, has put down something totally irrelevant to the subject." We have no doubt the butcher, wine-merchant, or tailor of this said gentleman would be disposed to question his mental condition if he so acted when they requested payment of their respective accounts. Again: "The *memory is the faculty at fault in such cases*, (moral insanity.)" Is the memory, we would ask Dr. Williams, a *moral* or *intellectual* faculty? Let him consult *Locke*, *Dugald Stewart*, *Browne*, or *Abercrombie*, before he replies to the question. In cases of moral insanity, according to our experience, and the experience of all authorities, the memory is generally active and tenacious. Viewing the whole of his account of moral insanity, we should consider it as applicable to dementia as to the affection he purports to delineate. In the chapter on "moral insanity," the following observation is made, *apropos* of what it is difficult to say. "*Stupid persons* often forget what they are talking about, (and some what they are writing about), even in the midst of conversation, and a more or less complete absence of thought is occasionally produced by a too protracted mental effort." Surely this is not intended as Dr. Williams' description of one morally insane? We should imagine not, because, in illustration of the remark, the author cites the cases of John Hunter and Dr. Wollaston! In deciding the question, whether an "alleged lunatic is fit to be intrusted to the care of himself, or capable of managing his own affairs," our readers will be gratified to hear that Dr. Williams "does not accord with those who place the *property first* and the *person after*." This announcement will perhaps remove any apprehensions which might exist in the public and professional mind as to the opinions of this distinguished authority upon so important a point.

To Dr. Williams we are indebted for having discovered a new form of insanity, hitherto undetected by the medical psychologist. He says that there "*is a form of insanity to which* BUTLERS *are becoming much exposed, and if, from loss of place or any other circumstance, the intoxicating draught is withheld, depression follows, and they then often commit suicide. I believe more butlers have recently, in London, terminated their existence by their own hands than any other class of individuals.*"* We should have been obliged to Dr. Williams if he had referred us to the statistical data from which he deduced this valuable conclusion. If a butler is discovered playing tricks with his master's wine—if he is detected, at unreasonable hours, flirting about the choice old port and madeira, and a necessity arises for his summary ejection from the wine cellar and the house, depression of mind, under such circumstances, will occasionally ensue; and it is possible that, like the celebrated cook, Vattel, the butler may commit suicide by cutting his throat with a piece of a broken wine-bottle: but we much question whether, if these unhappy accidents were to be of more frequent occurrence than they in reality are, we should be justified in introducing among our already too minute divisions and subdivisions of mental derangement a form of aberration to be called the "*Butler's Insanity.*" A cook burns his master's mutton —spoils his sauce—sends the salmon, turbot, or cutlet, half-dressed to table, and, in consequence of gross inattention to the duties of the *cuisine*, receives a peremptory notice to quit: should the unhappy cook run himself through with his own *spit*, or choke himself with a knuckle-bone, we doubt whether we should be warranted in talking of a form of lunacy to be denominated the "*Cook's Insanity.*" If we do so, we shall have in our psychological nosology the "*Kitchen-maid's Insanity,*" the "*Footman's Insanity,*" the "*Nursery-maid's Insanity.*" A painful necessity has recently arisen compelling us to intimate to our coachman the propriety of his immediately leaving the premises: the conse-

* Page 78.

quence may be, that, depressed by the consciousness of having lost our confidence, he may take a prolonged cold bath in the adjoining river Thames, and remain sufficiently long under the stream to induce asphyxia. Should this unhappily be the effect of his discharge from our service, our readers must not be surprised if in the next number of our Journal they find a chapter headed, "*A new Form of Lunacy—the Coachman's Insanity.*"

Proceeding onward, we find Dr. Williams denominating *Erotomania* to be a "*metaphysical* disorder," because "the sentiments" are "affected." A "metaphysical disorder," indeed! "Nymphomania, or satyriasis, arise (he says) from *physical* causes;" but we ask, is not this also the case with regard to the "metaphysical disorder," erotomania, as well as *every other form of insanity?* Dr. Williams suggests for the cure of erotomania, "a happy marriage." We question the utility of the remedy. "Erotomania" and "nymphomania" are both described in the chapter on Moral Insanity. Does the author consider these disorders as illustrations of that class of patients "who are insane in *conduct,* and not in *ideas?*" such being the definition which the author quotes with approval, of moral insanity. "One of the earliest indications of insanity *attacking women* is the change of ideas, sentiments, and actions."* Is this not also the case with *men?* The important subject of *puerperal insanity* is discussed in *thirty-four* lines. We cannot divine why the author should discuss this form of derangement in his chapter on Moral Insanity. It is surely out of place there? The only advice he ventures to give with regard to the *treatment* of this form of disturbed mind, is, that "these cases ought not to be sent to a *mad-house* (elegant phraseology in a work purporting to be a scientific production!) it being rare for puerperal mania to continue long, especially when early and promptly treated; but (continues the learned Doctor) if, after a month, the symptoms still continue, the pulse being very quick, change of residence and removal from home should not be generally longer postponed." Whilst thanking the author for these valuable suggestions, fraught with so much wisdom and sagacity, we may observe that he would have enhanced our obligation if he had informed us where the patient should be removed to? The chapter on "Dementia" is totally valueless. The whole subject of dementia and idiocy is dismissed in *five* pages, and contains nothing beyond an attempt, and a very laboured one it is, to define idiocy, fatuity, and dementia; the definitions of idiocy are taken from "Dr. Johnson's Dictionary," "Blackstone's Commentaries," and "Coke upon Lyttelton!" The preliminary observations of Dr. Williams, in his chapter on the "General Treatment of

* Page 81.

Insanity," contain a gross and inexcusable libel upon the profession. He says:

"It forms the exception for medical men to pay any attention to mental disease; and hence, when a case of insanity occurs in private practice, the individual so affected is either sent away at once to a lunatic asylum, or *the medical attendant, being himself alarmed, restrains his patient by violent measures.* The general ignorance of diseases of the mind, so prevalent throughout the profession, has frequently led to very unjust detentions; and if any medical man, so uninformed upon this subject, is requested to visit an alleged lunatic, he goes prepared to *prove* insanity; whereas his object should be to ascertain the exact state of the patient's mind, and to see whether there would be danger to life or property in allowing him personal freedom; but the very fact of seeing a person already manacled has, alas! been to many sufficient proof of his insanity; and, indeed, as Sir Henry Halford has said, if already confined, his condemnation is almost certain."—p. 90.

We maintain that it does not *necessarily* follow, as Dr. Williams asserts, that, if a patient be *not* sent to an asylum, "the medical attendant, being himself alarmed, *restrains his patient by violent measures.*" We deny the fact: the disposition is, we think, otherwise on the part of the profession. The *ultra* views on the subject of non-restraint, promulgated by a few over zealous members of the profession, have, in some cases, unhappily, led to the sacrifice of valuable lives. Again, upon what ground is Dr. Williams justified in saying that when "a medical man is requested to visit an alleged lunatic, he goes prepared to *prove* insanity?" We declare this to be a scandalous imputation upon the profession. Our readers will, no doubt, feel greatly obliged to Dr. Williams for informing them, that "no medical man is warranted in signing a certificate of a patient's unsoundness of mind *without having seen such patient.*" If he were to do so, contrary to the *express stipulations of the statute,* he would expose himself to an action for misdemeanour. Is the author of this work aware of the fact? We should suppose not, or he would not have offered *such advice* to the profession. After a fair proportion of twaddle about not listening to the "mere representations of friends"—that the "application of the family is no sufficient warrant for confinement;" that "personal observation alone can justify any medical man in signing a certificate of unsound mind"—Dr. Williams makes the subjoined grave accusation against his professional brethren:—"The *generality of medical men,* when asked to see a case, go with the *full intention* of establishing insanity, not to disprove it" (p. 91). Need we attempt a refutation of this calumnious statement? Perhaps Dr. Williams may yet have the satisfaction of hearing some distinguished member of the bar, or judge on the bench, *quote*

*this very passage* to establish, that the opinions of medical men relative to the subject of insanity are totally worthless. It is our duty to discountenance these attempts to depreciate the value of medical testimony, let them proceed from whatever quarter they may. It is, alas! mortifying to be compelled to repel an arrow aimed at the members of an honourable profession from the hand of one of our own brethren!

The passage we have just quoted and commented upon, is nothing more than a new and offensive edition of Lord Truro's unjustifiable observation, "that a medical man would give any opinion in lunacy that he was paid for;" an imputation which has been so severely animadverted upon by *all* the medical journals. "Medical men (says Dr. Williams) should never enter court as *partisans*." Of course not; "their object should be to establish truth;" (certainly.) "When examining a patient take care he is not agitated (sensible.) Gain his confidence (often a difficult object to attain) and endeavour to ascertain whether he has not been previously excited." The Doctor does not say *excited by what;* whether by the *disease*, his *medical attendants*, or "unprincipled relations." "The most monstrous means have been adopted (continues the author) to intimidate weak-minded individuals; and fraud, conspiracy, and intimidation must be met by perspicacious sagacity."—Right; but how few (according to this learned Theban) have the amount of "perspicacious sagacity" sufficient to overcome such a degree of base conspiracy. Dr. Williams communicates to the profession the important fact, "that a person improperly taken and detained as a lunatic, may maintain an action for assault." We trust this announcement will be consolatory to those of our readers who may be nervously apprehensive of the "mad doctor," the "medical certificate," or of being kidnapped, and confined by kind relatives in an asylum. It is very important that the members of our profession should, with these pains and penalties staring them in the face, have a clear conception of the *kind of patient* they are justified in depriving of liberty. Dr. Williams, conscious of the necessity of enlightening the profession upon this point, generously lays down rules for our guidance. We cannot sufficiently express our gratitude to the Doctor for his lucid instructions. He says, "There can be no doubt as to the necessity of placing under control a *furious maniac*, who would be constantly injuring himself or others." So far the advice is unexceptionable; but, fearful we might be led into error, and be disposed prematurely to interfere with the free agency of the Queen's subjects, the Doctor kindly develops still further his views upon the point, and adds, "and should he (the alleged lunatic), in addition, eat his own excrement, this would even render more surveillance and cleanliness necessary." So we should suppose. In the name of those associated with the treatment of the insane, we thank Dr. Williams for this extremely satisfactory advice. For the future, writs of *habeas corpus,* and actions for false imprisonment, will only be referred to as remnants of the dark ages. Let us clearly comprehend, "that there can be no doubt as to the necessity of placing under control a *furious maniac;*" but if our friends have any qualms of conscience in so acting, these will all disappear if, superadded to this, the "patient should eat his own excrement." "How often (says Dr. Williams) is a man sent to an asylum by his friends *because he is eccentric and irritable.*" We doubt the fact; nothing is easier than to make general statements and assertions of this kind; but as two medical men must certify not only as to the presence of insanity, but to such a *kind* and *degree* of insanity as to justify confinement, we are disposed to consider the occurrence of which the author speaks, *extremely rare;* in fact, we do not think it possible, considering the character of the members of our profession, the vigilance of the commissioners, and the amount of surveillance to which private asylums are, in the present day, subjected. After talking of an "eccentric" and "irritable" person being sent unjustly, by his friends, to an asylum, the author, with wonderful pathos, exclaims, "how dreadful for a patient ("only eccentric" and "irritable") just becoming conscious, with reason dawning upon him, to find himself in a mad-house." When speaking of the "eccentric" and "irritable" patient, the Doctor said nothing of the loss of consciousness and reason; surely, if these co-existed with the "eccentricity" and "irritability," a good and valid reason existed for placing the unhappy patient in a position most advantageous for recovery; and, instead of being appalled at finding himself in a "mad-house," after his restoration to consciousness and reason, one would imagine that he would be grateful to those who, in the hands of a wise Providence, had been instrumental in restoring to him the healthy exercise of mental faculties.

When talking of the effects of associating with the insane, the author observes, "that VERY FEW *nurses or keepers live under such exposure many years without themselves becoming insane!*" We never knew an instance corroborative of this assertion. The statement has no foundation in fact. If Dr. Williams doubt our word, let him ask those whose *practical experience* in the matter qualifies them to pronounce an opinion upon the point. "Moral treatment is more effective in the early weeks (of an attack of insanity), than at any subsequent period." This is contrary to the experience of *all* practical men. In the *early stages* of derangement, the *medical* treatment is the most essential and important, *because* the symptoms are generally more *acute* in their character, and clearly dependent upon *physical ill-health.* As the disease advances, and the bodily symptoms disappear, moral means are often of great service in the treatment of the insane. Dr. Williams suggests the propriety of removing "idiotic or highly eccentric persons, especially if noisy, from public gaze, in large towns, as the less such cases are exposed the fewer examples may reasonably be expected," (p. 108.) By what authority and power we are to remove "the eccentric persons" from "public gaze in large towns" the author has forgotten to inform us. The lunacy statute contains no clause justifying us in interfering with *eccentric* persons, however offensive they may be to the "public gaze," in small as well as "large towns." "Directly a person, be he rich or be he poor, entertains erroneous impressions, and often when only eccentric, *away he is hurried to an asylum,* where the chances of his cure are as remote as is the love which has, not unfrequently, *especially in the upper classes,* dictated his removal." We are puzzled which most to admire in the above paragraph—its elegance of literary diction, or the important truth which it developes. "Be he rich or poor," it matters little, if he have "*erroneous impressions,*" or is "*eccentric,*" his family, in the exuberance of their affection, "hurry him to an asylum," where, unhappily for the poor man, his "chances of recovery" are said to be "remote." The "*upper classes*" appear specially to merit our author's animadversion, for upon their heads he often opens the phials of his wrath. We would have "Belgravia" beware. We should have been thankful to Dr. Williams if he had deigned to have been a little more explicit on the subject of "erroneous impressions." If the existence of *these* be a justification for incarceration in a lunatic asylum, Dr. Williams must be on the look out! So forcibly impressed is the author of the great truth he has enunciated, that, in the very next paragraph, he again exclaims, "the mistake seems to be, that a person is considered a fit subject for a *lunatic asylum, merely* because he holds *fictitious or erroneous ideas,* and this applies both to the rich and the poor." We trust Dr. Williams does not feel unnecessarily alarmed for his own safety? The author appears to entertain, in common with many ignorant of the real character of the first class modern asylums, a horror of what he, with great want of taste, designates a "mad-house." In every chapter this feeling shows itself. "How dreadful for a patient to find himself in a mad-house." "There cannot be a doubt, that numbers, now the occupants of lunatic asylums, ought never to have been subjected to such treatment." After relating a case of recovery from an attack of delirium, he exclaims, "how different might the result have been, if placed (we suppose, the patient) within a lunatic asylum." "In an incipient case of mania, it is far better to treat it at the *patient's own house.*" We would add, particularly if the family should have the advantage of the author's skill and experience. "It is considered advisable, that, whenever a person's means will at all admit of his being treated *at home, that this* is always preferable." We might proceed *ad infinitum, usque ad nauseam,* in quoting analogous passages, embodying a wholesale and indiscriminate abuse of institutions for the treatment of the insane. But the selections we have made are sufficient to establish the *animus* as well as ignorance of the author. The reader having perused the previous passages, we would suggest that they should be taken in connexion with the following observations: "*Throughout Europe* (says Dr. Williams, p. 115), *physicians are agreed, that separation and seclusion are of the* GREATEST BENEFIT IN THE TREATMENT OF INSANITY." Again: "*Early seclusion is often of the greatest service.*" If "seclusion" does not mean confinement in an asylum, what idea are we to attach to it? Assuredly Dr. Williams does not recommend a recourse to that barbarous mode of "seclusion," termed the "*cottage system,*" of isolating the insane, which the Earl of Shaftesbury so eloquently denounced in the House of Commons, and which has been so frightfully and disgracefully abused?

If Dr. Williams prefer the snug cottage in St. John's-wood to a well-regulated private asylum — the irresponsible authority and management of a keeper, and *occasional* hurried visit of a medical man, to his kind, skilful and *daily* surveillance — we feel disposed to exclaim—may God protect the unhappy lunatic! If a patient be restored to sound mind under *such a system* of isolation, it will be *in defiance,* and not *in consequence,* of the means used for his restoration. We maintain *that it is impossible to carry into effect any* CURATIVE SYSTEM, *of either medical or moral treatment, in cases of actual insanity, outside the walls of a lunatic asylum.* In lodgings, at home, or in cottages, where the miserable patient must necessarily be left the greater portion of his time to the unlimited control and exclusive association of the attendants employed to watch him, the mind *rarely rallies from its disordered condition.* The chances of complete recovery in asylums are increased at least 50 per cent., and this will be obvious when we consider that in a well-regulated establishment the patient is night and day under the eye of the vigilant, anxious, and skilful physician; that the operation of the medicinal agents administered to promote recovery are carefully watched; that the patient has the advantage of the most approved medical treatment, and is subjected to the minimum amount of restraint; whilst, in lodgings or in cottages, the poor afflicted patient is generally either in a strait waistcoat or tied down to his chair, and is left day and night to the tender mercy of perhaps coarse and brutal attendants. The medical man visits his

FIG. 1.

FIG. 2.

New-Torturing Bed.

patient perhaps once or twice a week,—it may be *every day;*—but we ask those acquainted with the treatment of the affections of the mind, whether, under such a system of treatment, the probabilities of recovery are not very remote, if not entirely hopeless? We have seen patients subjected for *months* and *years* to this species of isolation of which Dr. Williams speaks, without making the slightest approach towards restoration to health; and yet these same patients have, after a few months' residence in a well-organized private asylum, been entirely cured. "An asylum," says this eminent psychologist, "*is at present* (what they *will* become, should Dr. Williams have one of *his own*, remains to be seen) *a necessary evil.*" What says the *insignificant* and *obscure* ESQUIROL. "An asylum," says M. Esquirol, is "an INSTRUMENT OF CURE, and, in the hands of a skilful physician, THE MOST POWERFUL THERAPEUTIC AGENT AGAINST MENTAL MALADIES." "A necessary evil," indeed! *Eating* and drinking are necessary evils; *sleeping* may be viewed in the same light. It is a "necessary evil" that we should be obliged to build houses, wear clothes, marry and have children, and even *print books.* If our first parents had not transgressed, and eat of the fruit "of that forbidden tree," and thus

"Brought death into the world, and all our woe,"

these things would have been quite superfluous. It is the *disease* which is the "*evil;*" the *means used for its cure* certainly cannot be so designated without a gross misuse and perversion of the Queen's English. "As there *must be lunatic asylums,*" says the author, "and AS (mark the acute logician!) *the majority of them are unfortunately densely thronged,* (why so?) *the importance of classification cannot be over-estimated,*" (p. 119.) Can our readers trace any connexion—necessary connexion—between the fact referred to in the *first* and the assertion contained in the *latter* part of this sentence? We might as well say, as there must be horticultural gardens at Chiswick every year, and as the next fête is likely to be "densely thronged," the importance of umbrellas cannot be over-estimated! We pray the attention of our readers to another lucid passage. "A man who has once been the occupant of a mad-house seldom regains his social position, and (mark the corollary!) *therefore* it is so essential to remove all predisposing causes; and first as to intermarriage."* The first assertion in this

* It would, indeed, be a sad and discouraging reflection, considering the amount of insanity, and the number of the patients under treatment, and discharged as "cured," from both public and private asylums, if there were the slightest pretence for Dr. Williams' bold assertion. We unhesitatingly deny the fact. We have before us the report of the "*Massachusetts State Lunatic Asylum,*" and in it we find Dr. Chandler, the physician, making the following remarks: "I have known a few individuals who were brought here insane, and who recovered to become better citizens than they were before. Their minds and feelings acquired strength and soundness by the disease, and by undergoing the process of cure, as some musical instruments are said to be improved by being paragraph contains a positive error. Hundreds and thousands who have been confined, and justly, properly, and kindly confined, in asylums, and that too for a considerable period, have regained their "social position." Need we, in confirmation of our opinion, appeal to the experience of men of great and established eminence? The fact is undeniable, indisputable—and it appears extraordinary how any man in his senses could doubt it. As a specimen of the author's brilliant literary attainments—of the classic dignity of his style—of the purity of his diction—the vividness of his fancy—we quote the subjoined passages in full. Shade of Burke, Addison, and Johnson, defend us!

"I am aware (important admission) it is said *mental disease is complicated—it is so* (nervous language); but there is no very great difficulty in estimating the amount of benefit resulting from any established rules of treatment which have generally hitherto been adopted. (We should imagine there was, after reading this work.) Disease of the mind is complicated, *and the persons who have specially undertaken to cure that disease have, at present, individually done very little in the way of suggesting either therapeutical, moral, or general means for alleviating or curing such an afflictive disorder, and this too with ample means of investigation before them; the desire has always been to keep the system or plan of treatment close. Even to this day their practice is often secret—empirical.*"

What consummate ignorance and impertinence! We will, upon *this* occasion leave the Doctor to the tender mercies of the English Psychologists, who may, and perhaps justly, flatter themselves that they have done *something* towards the advancement of our knowledge of the pathology of insanity, and who certainly are not open to the grave imputation of either a "close" or "empirical" "plan of treatment."

"Those *errors of society* which *every person must necessarily mix with* should be judiciously exposed, their evils shown; for if the mariner is previously made aware of the existence of the hidden rock, that is generally sufficient to prevent him from foundering upon it (not invariably so); at the same time *there are evil and wicked machinations and designs, which,* as *they but seldom expose themselves to public gaze,* and though miserably enslaving those still more miserable persons who are enslaved by them, are yet happily confined to the few, and those frequently only the offscum of society—therefore it is unwise, it is prejudicial to the best interests of individuals and of the public in general, to expose and propagate, even in the way of caution, the more refined broken and repaired again." Such is the experience of all engaged in the treatment of the insane. It is a fact that in some instances the judgment appears more vigorous, the affections more evenly balanced, the volition stronger *after* recovery than before the development of insanity. We readily admit that the mind cannot be subjected to frequent attacks of disorder without having its faculties impaired; but the assertion of the author that a man once having been confined in an asylum, "seldom regains his social position," is a perfectly *gratuitous,* and is in direct opposition to the experience of those whose practical opportunities for observation entitle them to form a sound and safe opinion upon the subject.

systems of vice, the more intensely devilish seductions of iniquity, and the more so, as no person ever can reach this climax (what climax ?) at once ; as there are numerous paths of virtue, so there are yet more numerous roads to vice, and few (few *paths* or *persons ?*) are so created as to become proficients at once; and therefore it is when sin is hurling down a young man headlong, (into an asylum ?) that the beacons should be brought (after he is down, or whilst being hurled ?) prominently before him to warn him of his danger."

After the reader has rallied from the overpowering effects of the glowing and impassioned eloquence of the above extract, let him ask himself what is meant by "*Those errors of society which every person must necessarily mix with,*" and which the author conceives should be "judiciously exposed ;" to what does Dr. Williams refer when he speaks of the "*evil and wicked machinations and designs, which, as they seldom expose themselves* (we presume the fear of the police would deter them from doing so) *to public gaze, &c.?*" We should imagine that "evil and wicked machinations and designs" would find some difficulty in "exposing themselves to public gaze," therefore we ask, is not the worthy Doctor rather severe in his animadversions ? We will not venture to make any further analysis of this psychological paragraph. It is certainly a fine specimen of pure and classic English composition. The only doubt we entertain is, whether it is not *borrowed* from the *Spectator.*

We had marked several other passages, equally sublime, for quotation, but we have already exceeded our limits. We shall content ourselves with one more extract. Dr. Williams observes, in a paragraph which has no reference to the one immediately preceding, "*light is the only effect the moon has upon lunatics : they cannot sleep, the moon is at the full.*" If the author had sufficient knowledge of literary composition to explain in unadorned English the notion he wished to convey, he could easily have developed the idea struggling,

"Like the pale moon's misty light,"

for existence, in the sentence just referred to. It is not difficult (obscure as the passage is) to divine Dr. Williams' meaning. He denies that the lunar rays have, as many suppose, a *specific influence upon the insane;* whatever the effects may be, they are entirely owing to the *action of light,* which interferes with the sleep of the insane, particularly when the light is intense, as it is when the moon is at the full. We merely record the views of the author, without giving any opinion of our own upon the point. It has given us much pain to be obliged, in duty to our numerous readers, to speak in such disparaging terms of Dr. Williams' work. The author requires to be taught the necessary lesson, that it is the duty of men to *learn* before they attempt to *teach,* and that without long experience and great sagacity no man can by a hop, skip, and a jump, place himself in the professor's chair. In a medical point of view, the essay is of no value; as a piece of literary composition, it is, we regret to say, contemptible. The author appears to have sat down to write his book without an idea of his subject, (beyond what he has found in the text books), or of the arrangement necessary for its lucid exposition. On several occasions, his fancy has taken an elevated flight; and in more than one ambitious attempt at *fine writing*, he has lost himself among a number of extravagantly hyperbolic expressions, reminding us of the passage descriptive of the "fix" into which a poor poet had placed himself in his vain effort to convey to his readers his exalted conception of the sublimity of an American forest:—

"When I hear the gentle breeze,
A blowin' in among the trees,'
I can't my thoughts in words express,
But they are mighty strong,—nevertheless."

## Figures and Tables

**FIG. 1. FIG. 2. f1:**